# Design and Simulation of an Electron Optical System for Terahertz Vacuum Devices

**DOI:** 10.3390/mi16080928

**Published:** 2025-08-13

**Authors:** Muhammad Haris Jamil, Zhiwei Lin, Hamid Sharif, Nazish Saleem Abbas, Wenlong He

**Affiliations:** 1 College of Electronics and Information Engineering, Shenzhen University, Shenzhen 518060, China; 2 College of Physics and Optoelectronics, Shenzhen University, Shenzhen 518060, China

**Keywords:** sheet electron beam, periodic cusped magnet, electron optic system

## Abstract

An electron optic system (EOS) consisting of a sheet electron beam gun (SEB) and a pole offset periodic cusped magnet (PO-PCM) is reported for 340-GHz frequency. A sheet electron beam with a voltage of 29 kV, beam compression ratio of 16, and a beam waist of size 0.17 mm × 0.044 mm was designed and optimized using computer simulation technology (CST). The EOS was capable of transmitting the beam with a current of 6.9 mA through a beam tunnel of size 0.516 mm × 0.091 mm, having a length of 60 mm with the help of a pole offset periodic cusped magnet. The axial magnetic field generated by the PCM was 0.32 T. The EOS was efficient enough to transmit the beam stably through the beam tunnel with a transmission rate of 100%.

## 1. Introduction

Vacuum electronic devices (VEDs) are widely used in commercial and military applications requiring high power at high frequency [1,2,3,4]. A core element of a VED is the electron optic system (EOS), which consists of an electron gun and a magnetic focusing structure. Leading to the limitation on the output power, conventional circular beam gun devices do not fulfill the demand of producing high-power coherent radiation at terahertz frequencies. Therefore, the sheet electron beam (SEB) gun devices come to the rescue.

The critical issue in realizing the sheet beam vacuum devices is the generation and transportation of the SEBs, especially at high operating frequencies. This is because the high operating frequency inevitably employs a tiny beam tunnel, which would raise the risk of electron interception in the beam tunnel [5]. Moreover, the SEB with a high current density is necessary for a device working at the millimeter–wave and terahertz–wave, resulting in a huge space charge force, which will bring great difficulty in focusing [6]. To deal with the dilemma, one way is to design an electron gun with a high compression ratio [7,8]. This will increase the current density of the beam and improve the output power of the device directly. Another way is to increase the length of the beam tunnel under the condition of a high electron transmission rate [9,10] so that the beam can have more opportunity to interact with the electromagnetic wave, thus improving the gain, output power and the electron efficiency of the device.

An external magnetic field is used to confine the sheet beam within the tunnel without being intercepted. To avoid the instabilities [6,11], a periodic rather than uniform guiding magnetic field was proposed. Due to miniaturization configuration, PCM has been successfully applied to vacuum electronic devices employing SEB. The PCM focusing is preferable to the terahertz TWTs, where the transverse dimension of the interaction circuit is very limited, and even a small horizontal swing of the beam cannot be tolerated.

Various studies have been conducted that deal with the challenges of designing EOS at terahertz (THz) frequency, out of which a few are cited in the references. For example, in [12], a traveling wave tube with sheet electron beam operating at 263 GHz was discussed, which used PCM–tunable quadrupolar magnet (PCM–TQM) to transport a 19 kV, 0.15 A sheet beam through a tunnel of length 45 mm. The transmission rate was measured to be 97.6% with the RF signal. Yiyang Su et al. in [13] reported an electron optic system working at 0.22 THz frequency. The beam, having a beam voltage of 16.5 kV and a current of 0.5 A, is transported successfully through 18 mm long tunnel using an approximately 0.6 T uniform magnetic field.

In [14,15], TWT amplifiers were discussed at a frequency of 340 GHz. The EOSs used were comprised of sheet electron beam and periodic magnetic structures, having magnetic field values of 0.32 T and 0.46 T, respectively. The SEB in [14] has a voltage of 19.5 kV and a current of 60 mA, while in [15], the SEB used comprised a 15 kV voltage and 25 mA current. Both designs were able to transport the electron beam with 100% transmission rate. The overall output power of the tubes was 25 W and 3.17 W, respectively.

This article presents an electron optic system with a good beam compression ratio and long beam channel length designed for 340 GHz frequency. The device operates at 29 kV and generates a 6.9 mA sheet electron beam, which could be transmitted stably over a length of 60mm with the help of a periodic axial magnetic field of 0.32 T. The current value as low as reported in this article reduces the space charge effect and improves the bunching (interaction of e-beam and RF signal) in the high frequency structure, hence giving more uniform gain for the wider frequency band. Low current also reduces the thermal stress and boosts the cathode life. The voltage of 29 kV improves coupling with the high-frequency structure, therefore providing maximum energy transfer to the RF signal. The organization of this paper is as follows. In Section 2, we will discuss the design and simulation results of the SEB gun. Section 3 discusses the design and fabrication results of the PCM structure. Co-simulation and transmission properties of EOS are discussed in Section 4, and the conclusion is presented in Section 5.

## 2. Sheet Electron Beam—Design and Simulation

**Figure 1:**
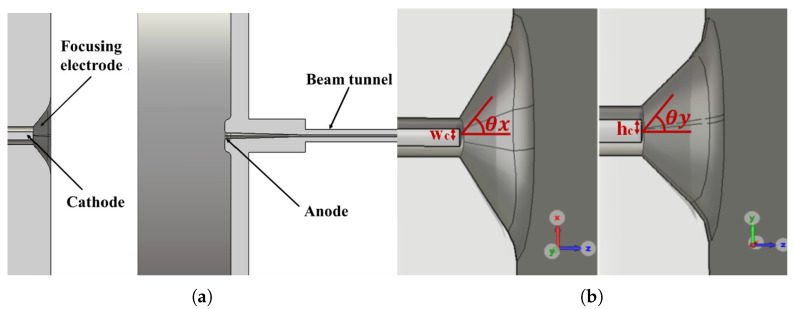
(**a**) Electron gun; (**b**) left: x–z (wide side), right: y-z (narrow side) plan views of the emitter and focusing electrodes.

Based on the design of the Pierce-type gun [16], an electron gun was designed in this study. The gun consisted of an elliptical cathode, a beam-focusing electrode and the anode with the beam tunnel, as shown in Figure 1. The electron gun parameters, including 29 kV operating voltage, a 6.9 mA beam current, and a compact 0.516 × 0.091 mm beam channel, were specifically optimized for integration with our 340 GHz high-frequency system, which constitutes the next stage of this research project. The output power of the beam gun was expected to be around 200 W.

An SEB gun was designed based on the above-mentioned parameters using computer simulation technology (CST). The dimensional values of the gun are given in Table 1, where $w_{c}$ is the half width and $h_{c}$ is the half height of the cathode, respectively, $\theta_{x}$ and $\theta_{y}$ are the beam-focusing angles in the xoz-plane and yoz-plane, respectively. $w_{t}$ is the width of the tunnel, and $h_{t}$ is the height of the beam channel. The cathode of size 0.31 mm × 0.39 mm and focusing electrodes were biased at −29 kV, and the anode was grounded (0 V). The electrostatic trajectory of the beam was studied using a particle tracking solver, and the simulation was conducted under the limitation of space charge emission. The mesh size of the model was more than 20 million, and the number of emission points on the cathode were 2646.

**Figure 2:**
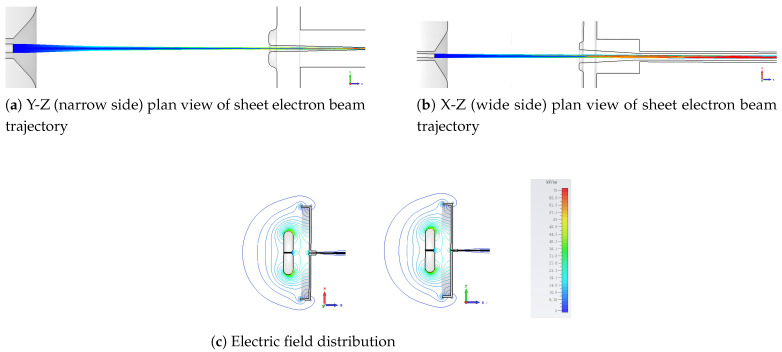
Beam trajectory and electric field distribution: (**a**) Y-Z plane, (**b**) X-Z plane, and (**c**) electric field in both planes.

The cathode emits electrons that move in the form of a beam towards the anode. The current of the emitted beam was 6.9 mA as depicted by the simulation results, making the cathode current density 7.4 A/${\mathrm{cm}}^{2}$. The focusing electrode was used to converge the electron beam into an approximately elliptical shape. The anode has a rectangular opening slit that allows the beam to pass through. Thus, the beam converged near the anode opening and then passed through the beam channel towards the collector. The simulated beam trajectory and the electric field distribution of the e–gun are shown in Figure 2. The electric field value at the cathode and the focusing electrode was approximately equal to 62 kV/cm.

**Figure 3:**
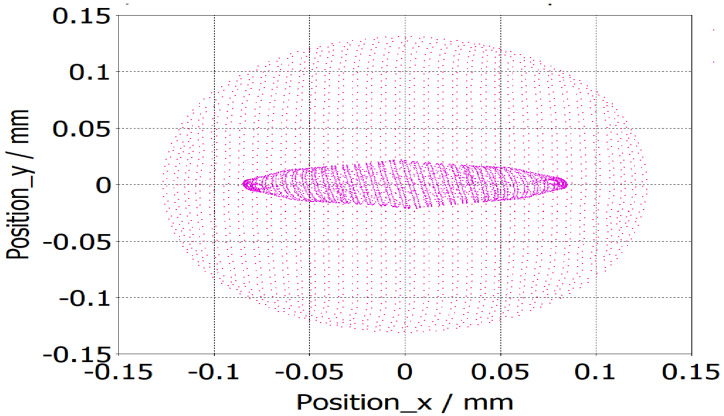
Beam cross-section (red) at cathode and beam waist (pink).

**Figure 4:**
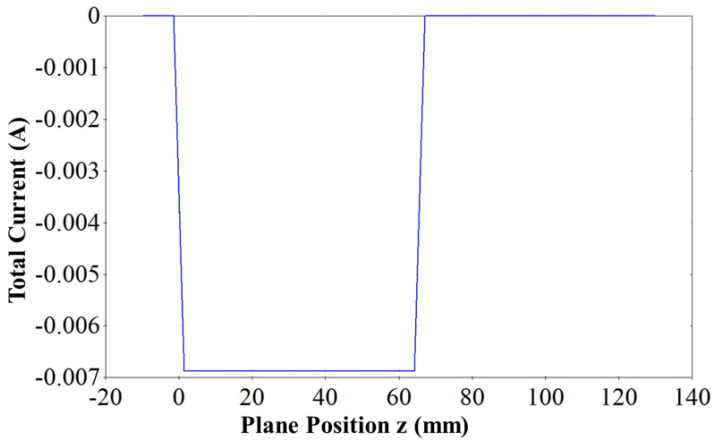
Total beam current.

Simulation results showed that the size of the beam at the beam waist was 0.17 mm × 0.044 mm. This made the overall compression ratio M of the beam approximately equal to 16. The elliptical shape of the beam at the beam waist is shown in Figure 3, making it admissible for the beam wave interaction. The current density of the beam was calculated to be 117 A/${\mathrm{cm}}^{2}$. A low-current electron beam, as shown in Figure 4, offers significant advantages, including enhanced beam laminarity, better RF interaction, reduced space charge effects, and lower thermal load, increasing device longevity and energy efficiency.

## 3. Design and Simulation of Pole Offset Periodic Cusped Magnet

The electron beam, after passing the beam waist, starts to diverge due to the space charge effect. To confine the beam inside the beam tunnel, an external magnetic field was required. The pole offset–periodic cusped magnetic (PO–PCM) structure was used for this purpose. The transmission of a sheet beam under the PO–PCM satisfies the following expressions.

(1a) $$\begin{array}{cc} m_{o}\frac{d\dot{x}}{dt} & =qE_{\mathrm{sc},x}+q\left(\dot{y}B_{z}-\dot{z}B_{y}\right) \end{array}$$

(1b) $$\begin{array}{cc} m_{o}\frac{d\dot{y}}{dt} & =qE_{\mathrm{sc},y}+q\left(\dot{z}B_{x}-\dot{x}B_{z}\right) \end{array}$$

where $m_{o}$ is the rest mass of the electron and *q* is the charge of the electron, $E_{\mathrm{sc},x}$, $E_{\mathrm{sc},y}$ are the components of the space charge field and $B_{x}$, $B_{y}$ and $B_{z}$ are the components of the focusing magnetic field. $B_{z}$ is used to balance the space charge force in the y-direction (narrow), and $B_{y}$ is used to balance the force in the x-direction (wider).

The period of the magnetic structure is $L_{m}$, and $B_{o}$ is the peak value of axial magnetic field $B_{z}$. $L_{m}$ should be well smaller than the plasma oscillation wavelength ≥p typically ratio $\frac{L_{m}}{\lambda_{p}}<0.33$. To confine the SEB in the Y-direction, the magnetic amplitude $B_{o}$ of the PCM field should range from $1.5B_{bri}$ to $2B_{bri}$ of the Brillouin value, as given in [17].

(2) $$B_{bri}=\sqrt{\frac{1}{\gamma }\cdot \frac{\sqrt{2}I_{o}}{w_{b}h_{b}\epsilon_{o}\eta^{3/2}U^{1/2}}}$$

where γ is the relativistic constant, $w_{b}$ is the half width of the beam, $h_{b}$ is the half height of the beam, η is the charge-to-mass ratio of an electron, and *U* is the voltage of the beam. Another restriction between $B_{o}$ and periodic length $L_{m}$ is [18].

(3) $$\left(\frac{\eta B_{o}^{2}L_{m}^{2}}{U}\right)\leq \frac{417}{4}$$

To produce the Lorentz force in order to balance the space charge force in the x-direction, $B_{y}$ should satisfy the following conditions

(4) $$-eE_{\mathrm{sc},x}\leq \left|-ev_{z}\times B_{y}\right|$$

where $v_{z}$ denotes the electron velocity in the z-direction. Based on the above analysis, a pole offset periodic cusped magnet was designed and optimized using the CST magnetostatic solver, as shown in Figure 4. The mesh size used for simulation was more than 16 million. The pole pieces were set as pure iron with a nonlinear B-H relationship. The magnetic blocks used for the simulation have remanence of 1.1 T and coercive force of 833 kA/m. Using the half-height and half-width of the beam, which were 0.022 mm and 0.085 mm, respectively, along with the other parameters discussed in Section 2, the Brillouin magnetic field was calculated to be 0.2 T. The period of the magnetic system $L_{m}$ was 3.6 mm. The PCM structure consisted of a magnetic block and an iron pole piece with dimensions $w_{m}\times h_{m}\times d_{m}$ and $w_{p}\times h_{p}\times d_{p}$ having identical values of 15 mm × 7 mm × 1.8 mm was simulated. The value of the axial magnetic field $B_{z}$ that best matched the beam was found to be 0.32 T, which is 1.6 times the Brillouin magnetic field. The staggered distance of pole–piece in the x-direction $D_{X}$ was 3 mm. The distance between the upper and lower halves of the PCM was 3mm, and the length of the structure was kept around 100 mm just for experimental purpose.

**Figure 5:**
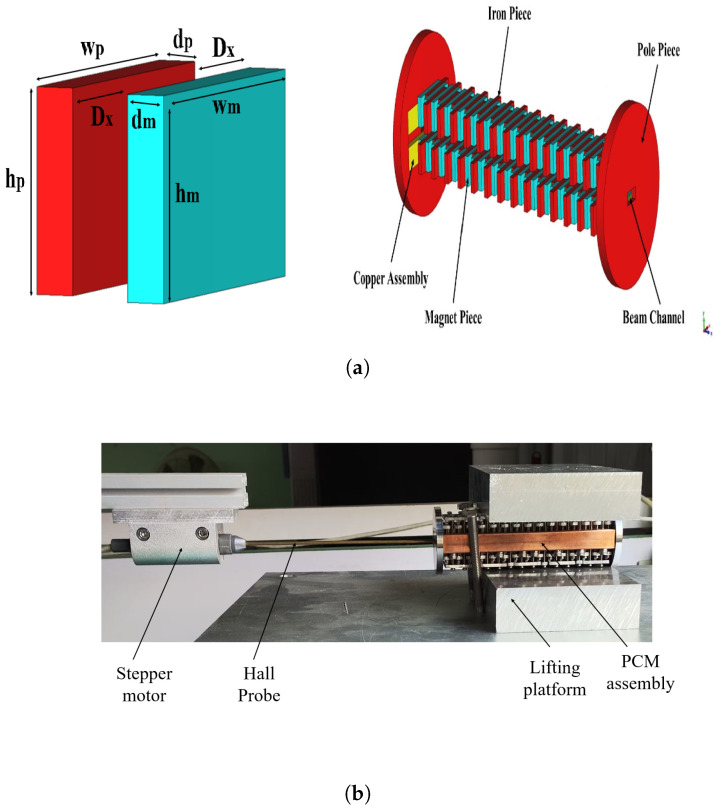
(**a**) PCM assembly CST model. (**b**) PCM assembly testing platform.

**Figure 6:**
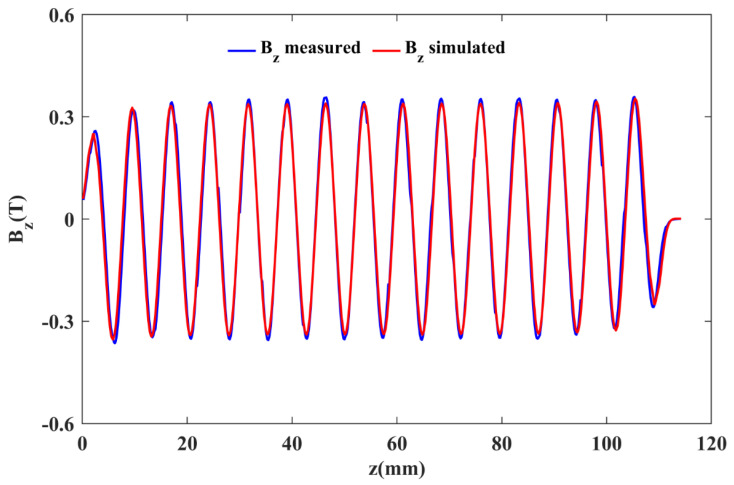
Graph of $B_{z}$ verses *z* axis.

To verify the simulation design, the above-discussed PCM structure with the mentioned parameters was physically fabricated. The magnetic material used was Samarium Cobalt, and the DT8A iron was used as the pole piece. Figure 5a,b shows the fabricated assembly of the periodic permanent magnet and testing platform used for the measurements. The results showed that the PCM focusing structure could provide a periodic $B_{z}$ of ~0.32 T. A graphical comparison between the simulated and experimentally measured data was made using MATLAB R2023a. The $B_{z}˘z$ graph in Figure 6 shows a good agreement between the compared values of $B_{z}$.

**Figure 7:**
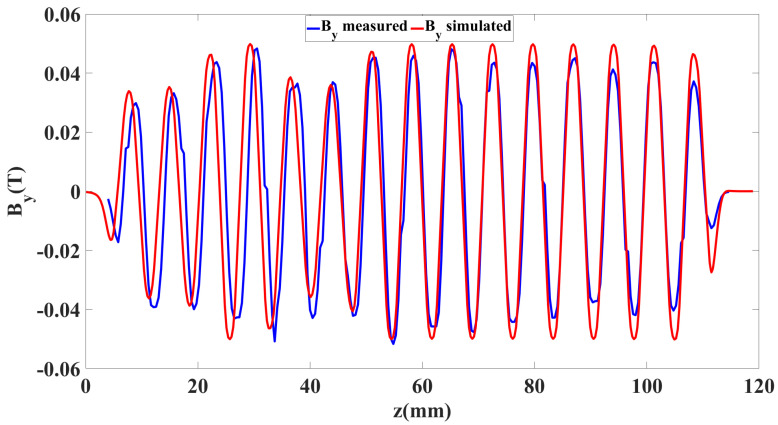
Graph of $B_{y}$ verses *z* axis.

$B_{y}$, which was necessary to confine the beam into the tunnel in a wider direction, was also measured and compared in the MATLAB graph, as shown in Figure 7. Since $B_{y}$ is sensitive to the vertical position of the Hall probe, a little deviation in the y-direction changes $B_{y}$ significantly. Figure 7 shows the measured $B_{y}$ and simulated $B_{y}$ at (0.2 mm, 0.18 mm), which are consistent. The numerical value of the simulated $B_{y}$ was 0.05 T.

Thermal expansion due to RF heating or beam interception in high frequency structure adversely affects the alignment of pole pieces and weakens the effectiveness of the transverse magnetic field. Such a degradation can be mitigated by active cooling and thermal shielding.

## 4. Electron Optic System and Transmission Property of SEB

**Figure 8:**
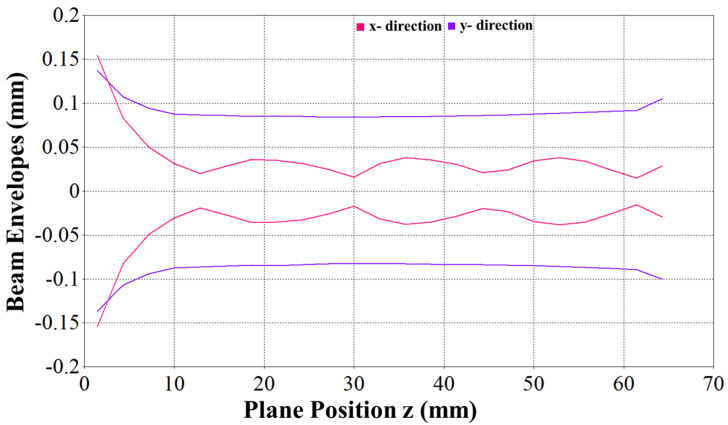
Beam envelope in narrow (*x*) and wide (*y*) directions.

An electron optic system is discussed in this section. EOS was composed of a sheet beam e-gun and a periodic magnetic focusing structure designed above. The calculated magnetostatic field of the simulated magnetic structure was imported to the sheet beam simulation model. One of the crucial problems during integration is the matching between the e-gun and the periodic focusing structure. The beam should enter the magnetic field at the beam waist, which was 12.8 mm away from the cathode. After importing the external magnetic field, the sheet beam e-gun model was simulated using the particle tracking solver. The beam envelope observed from the narrow side and wide side is shown in Figure 8. The simulation results show that the beam is confined well inside the dimensions of the beam tunnel, hence achieving a 100% transmission rate for a 60 mm long beam tunnel, as shown in Figure 4.

## 5. Conclusions

This paper presents the design and optimization of a periodic cusped magnet-focused sheet beam optical system for application in a 340 GHz traveling wave tube amplifier, representing the next phase of our research project. The simulated design achieved a beam current density of 117 A/${\mathrm{cm}}^{2}$ with a 100% transmission rate for a sheet beam having a voltage of 29 kV, a current of 6.9 mA and a beam waist of 0.17 mm × 0.044 mm. A PCM with an axial magnetic field of 0.32 T was used to transmit the beam through a relatively long beam tunnel of length up to 60 mm. The simulated PCM structure was fabricated, and the $B_{z}$ and $B_{y}$ magnetic fields were measured. Although slight variations were observed in the measured $B_{y}$, the axial magnetic field $B_{z}$ closely matched the simulated results, confirming the accuracy of the model. The device dimensions are compatible with standard microfabrication techniques, including CNC machining and LIGA processes. Furthermore, the optimized electric field distribution across the emitter, combined with an achievable current density of 117 A/${\mathrm{cm}}^{2}$, which is well within the practical operating range for scandate cathodes, enhances the design’s real-world feasibility.

## Figures and Tables

**Table 1:** Dimensional values of the sheet beam gun.

$w_{c}$	$h_{c}$	$\theta_{x}$	$\theta_{y}$	$w_{t}$	$h_{t}$
0.155 mm	0.195 mm	35.5°	30.5°	0.516 mm	0.091 mm

## Data Availability

The data that support the findings of this study are available from “the corresponding author” upon reasonable request.
